# Supplementary material for: The physical profile of female cricketers: An investigation between playing standard and position
Source: PLoS One. 2024 Jun 10;19(6):e0302647. doi: 10.1371/journal.pone.0302647 (PMC11164355; doi:10.1371/journal.pone.0302647)
Supplement: S1 Table — Cm = centimetres; kg = kilograms; mm = millimetres; n/a = non-applicable. (DOCX) [file pone.0302647.s001.docx]

**Supplementary Material One**: Individual playing and anthropometric testing results. Cm = centimetres; kg = kilograms; mm = millimetres; n/a = non-applicable

| Participant | Age (years) | Height (cm) | Weight (kg) | Position | Status | Sum of 8 Skinfolds  (mm) |
| --- | --- | --- | --- | --- | --- | --- |
| 1 | 24 | 165 | 66 | Non-seam bowler | Non-elite | n/a |
| 2 | 28 | 161 | 54.6 | Non-seam bowler | Elite | 59.2 |
| 3 | 23 | 164 | 61 | Non-seam bowler | Non-elite | n/a |
| 4 | 30 | 174 | 74 | Seam bowler | Elite | n/a |
| 5 | 30 | 168 | 61 | Non-seam bowler | Elite | n/a |
| 6 | 23 | 179 | 65.2 | Seam bowler | Elite | n/a |
| 7 | 31 | 168 | 69 | Non-seam bowler | Elite | n/a |
| 8 | 25 | 165 | 58.1 | Non-seam bowler | Elite | n/a |
| 9 | 27 | 169 | 66.8 | Non-seam bowler | Elite | 88.8 |
| 10 | 24 | 171.1 | 59.5 | Non-seam bowler | Elite | n/a |
| 11 | 26 | 160 | 53.8 | Non-seam bowler | Elite | n/a |
| 12 | 20 | 177 | 65.7 | Non-seam bowler | Elite | 114.4 |
| 13 | 30 | 165 | 55 | Non-seam bowler | Non-elite | n/a |
| 14 | 26 | 164 | 64.9 | Seam bowler | Elite | n/a |
| 15 | 23 | 173 | 69.1 | Seam bowler | Elite | n/a |
| 16 | 23 | 165 | 60 | Non-seam bowler | Non-elite | n/a |
| 17 | 25 | 168 | 60.2 | Seam bowler | Elite | n/a |
| 18 | 30 | 175.8 | 69 | Non-seam bowler | Elite | 106.5 |
| 19 | 25 | 173 | 65.2 | Seam bowler | Elite | n/a |
| 20 | 23 | 160 | 63.8 | Non-seam bowler | Elite | n/a |
| 21 | 26 | 165 | 60.1 | Non-seam bowler | Elite | n/a |
| 22 | 27 | 174 | 81.9 | Non-seam bowler | Elite | n/a |
| 23 | 30 | 159 | 61.5 | Non-seam bowler | Elite | n/a |
| 24 | 23 | 167 | 68.3 | Seam bowler | Elite | n/a |
| 25 | 15 | 161 | 50.7 | Seam bowler | Non-elite | n/a |
| 26 | 28 | 170 | 61.5 | Non-seam bowler | Elite | n/a |
| 27 | 20 | 166 | 53.1 | Non-seam bowler | Non-elite | n/a |
| 28 | 24 | 163 | 53.5 | Non-seam bowler | Non-elite | n/a |
| 29 | 30 | 164 | 76 | Seam bowler | Elite | 147.4 |
| 30 | 27 | 167 | 60.3 | Non-seam bowler | Non-elite | n/a |
| 31 | 30 | 162 | 57.6 | Seam bowler | Elite | 91.4 |
| 32 | 29 | 171 | 64.7 | Seam bowler | Elite | n/a |
| 33 | 21 | 172.45 | 72.8 | Non-seam bowler | Elite | n/a |
| 34 | 20 | 166.5 | 46.6 | Non-seam bowler | Non-elite | n/a |
| 35 | 30 | 165 | 71.8 | Non-seam bowler | Non-elite | n/a |
| 36 | 30 | 153 | 60.2 | Seam bowler | Elite | n/a |
| 37 | 21 | 164 | 70.4 | Seam bowler | Non-elite | n/a |
| 38 | 21 | 160 | 49.3 | Non-seam bowler | Non-elite | n/a |
| 39 | 17 | 172 | 70.3 | Seam bowler | Non-elite | n/a |
| 40 | 26 | 170 | 69.8 | Seam bowler | Elite | n/a |
| 41 | 20 | 172 | 62.6 | Seam bowler | Non-elite | n/a |
| 42 | 21 | 163 | 54.4 | Seam bowler | Non-elite | n/a |
| 43 | 17 | 172 | 62.6 | Seam bowler | Non-elite | n/a |
| 44 | 22 | 161 | 57.1 | Non-seam bowler | Elite | 117 |
| 45 | 22 | 176 | 75.1 | Seam bowler | Elite | n/a |
| 46 | 21 | 175 | 62 | Seam bowler | Non-elite | n/a |
| 47 | 22 | 180 | 72 | Seam bowler | Non-elite | n/a |
| 48 | 27 | 173.5 | 72.3 | Seam bowler | Elite | 114.2 |
| 49 | 21 | 171.5 | 64.8 | Seam bowler | Non-elite | n/a |
| 50 | 21 | 161 | 68.4 | Non-seam bowler | Non-elite | n/a |
| 51 | 30 | 160 | 60 | Non-seam bowler | Elite | 75.2 |
| 52 | 28 | 174.3 | 66 | Seam bowler | Elite | 98.5 |
| 53 | 24 | 162 | 55.8 | Seam bowler | Elite | n/a |
| 54 | 25 | 161 | 65 | Seam bowler | Non-elite | n/a |
