# Supplementary material for: The physical profile of female cricketers: An investigation between playing standard and position
Source: PLoS One. 2024 Jun 10;19(6):e0302647. doi: 10.1371/journal.pone.0302647 (PMC11164355; doi:10.1371/journal.pone.0302647)
Supplement: S2 Table — m = metres; s = seconds; CMJ = countermovement jump; IMTP = isometric mid-thigh pull; cm = centimetres; N = newtons; BW = bodyweight. (DOCX) [file pone.0302647.s002.docx]

**Supplementary Material Two:** Individual best result for each participant across physical capacity testing. m = metres; s = seconds; CMJ = countermovement jump; IMTP = isometric mid-thigh pull; cm = centimetres; N = newtons; BW = bodyweight

| Participant | Yo-yo (m) | 10m (s) | 20m (s) | Run2 Dominant  (s) | Run 2  Non-Dominant (s) | CMJ (cm) | Broad Jump (cm) | IMTP peak Force  (N) | Relative  IMTP  (BW) |
| --- | --- | --- | --- | --- | --- | --- | --- | --- | --- |
| 1 | 840 | 1.87 | 3.42 | 6.86 | 6.86 | 29.3 | 229 | 2896 | 4.47 |
| 2 | 1440 | 1.87 | 3.25 | 6.53 | 6.56 | 42.8 | 233 | 2299 | 4.29 |
| 3 | n/a | n/a | n/a | n/a | n/a | 31 | 186 | 2435 | 4.07 |
| 4 | 1120 | 1.9 | 3.39 | 6.87 | 6.91 | 37.8 | 182 | 2875 | 3.96 |
| 5 | 1080 | 1.97 | 3.4 | 6.7 | 6.84 | 29.6 | 204 | 2221 | 3.71 |
| 6 | 920 | 2.02 | 3.43 | 6.9 | 7.07 | 30.3 | 183 | 2360 | 3.69 |
| 7 | 880 | 1.86 | 3.22 | 6.71 | 6.71 | 34.8 | 239 | 2497 | 3.69 |
| 8 | 960 | 1.97 | 3.43 | 7.02 | 7.22 | 31 | 167 | 2076 | 3.64 |
| 9 | 1240 | 1.86 | 3.18 | 6.31 | 6.34 | 52.1 | 245 | 2359 | 3.60 |
| 10 | 1840 | 1.84 | 3.18 | 6.52 | 6.6 | 40.7 | 228 | 2077 | 3.56 |
| 11 | n/a | n/a | n/a | n/a | n/a | 34.8 | 197 | 1871 | 3.55 |
| 12 | 1480 | 2.08 | 3.56 | 7.08 | 7.17 | 32 | 200 | 2276 | 3.53 |
| 13 | 1080 | 1.9 | 3.28 | 6.84 | 6.95 | 36.3 | 220 | 1878 | 3.48 |
| 14 | 1000 | 1.9 | 3.25 | 6.72 | 6.72 | 41.8 | 209 | 2203 | 3.46 |
| 15 | 720 | 1.94 | 3.36 | 6.92 | 6.95 | 35.6 | 180 | 2340 | 3.45 |
| 16 | 1640 | 2 | 3.46 | 6.7 | 6.7 | 36.3 | 253 | 2005 | 3.41 |
| 17 | 1160 | n/a | n/a | n/a | n/a | 32.6 | 205 | 2002 | 3.39 |
| 18 | 1680 | 2.03 | 3.46 | 6.52 | 6.73 | 31.8 | 203 | 2293 | 3.39 |
| 19 | 1080 | 2.26 | 3.75 | 7.07 | 7.07 | 28 | 175 | 2149 | 3.36 |
| 20 | n/a | n/a | n/a | n/a | n/a | 33.7 | 200 | 2076 | 3.32 |
| 21 | 1080 | 1.95 | 3.44 | 7.15 | 7.18 | 35.1 | 203 | 1941 | 3.29 |
| 22 | 1000 | 1.93 | 3.36 | 6.69 | 6.69 | 38.6 | 256 | 2584 | 3.22 |
| 23 | 1480 | 1.9 | 3.23 | 6.5 | 6.53 | 38.7 | 219 | 1906 | 3.16 |
| 24 | 880 | 1.9 | 3.32 | 6.91 | 7.06 | 34 | 194 | 2115 | 3.16 |
| 25 | 880 | 1.91 | 3.4 | 6.91 | 7.09 | 37.2 | 191 | 1568 | 3.15 |
| 26 | 1400 | 1.99 | 3.42 | 7.02 | 7.03 | 33.7 | 196 | 1834 | 3.04 |
| 27 | 720 | 1.93 | 3.33 | 6.83 | 6.83 | 39.3 | 240 | 1582 | 3.04 |
| 28 | 1480 | 1.87 | 3.46 | 7.02 | 7.02 | 32.4 | 213 | 1593 | 3.04 |
| 29 | 1160 | 2.01 | 3.55 | 7 | 7.06 | 32.8 | 185 | 2252 | 3.02 |
| 30 | 960 | 1.89 | 3.29 | 6.76 | 6.76 | 35.6 | 247 | 1736 | 2.93 |
| 31 | 960 | 1.88 | 3.35 | 6.87 | 6.92 | 36.5 | 203 | 1577 | 2.79 |
| 32 | 720 | 1.87 | 3.26 | 6.67 | 6.73 | 32.8 | 187 | 1768 | 2.79 |
| 33 | 840 | 2.05 | 3.51 | 7.31 | 7.56 | 27.1 | 180 | 1961 | 2.75 |
| 34 | 1120 | 1.92 | 3.4 | 6.95 | 7 | 31.6 | 177 | 1223 | 2.68 |
| 35 | n/a | 2.21 | 3.7 | 7.15 | 7.32 | 29.1 | 174 | 1846 | 2.62 |
| 36 | 520 | 1.97 | 3.32 | 7.22 | 8 | 41 | 240 | 1542 | 2.61 |
| 37 | 880 | 1.92 | 3.41 | 6.88 | 6.9 | 34 | 180 | 1799 | 2.60 |
| 38 | 520 | 2.14 | 3.78 | 7.74 | 8.01 | 23 | 141 | 1253 | 2.59 |
| 39 | 640 | 1.99 | 3.47 | 7.14 | 7.25 | 36.9 | 170 | 1782 | 2.58 |
| 40 | 1040 | 1.94 | 3.36 | 6.83 | 6.92 | 35.1 | 200 | 1759 | 2.57 |
| 41 | 440 | 2 | 3.52 | 7.09 | 7.12 | 31 | 187 | 1574 | 2.56 |
| 42 | 600 | 2.09 | 3.49 | 7.02 | 7.12 | 35.3 | 170 | 1367 | 2.56 |
| 43 | n/a | 1.95 | 3.43 | 6.97 | 7 | 35.6 | 154 | 1509 | 2.46 |
| 44 | 880 | 1.98 | 3.43 | 7.04 | 7.05 | 29 | 201 | 1357 | 2.42 |
| 45 | 1160 | 2.05 | 3.56 | 7.15 | 7.39 | 26.7 | 166 | 1770 | 2.40 |
| 46 | 840 | 1.94 | 3.43 | 7.07 | 7.08 | 31.5 | 183 | 1431 | 2.35 |
| 47 | 880 | 1.89 | 3.27 | 6.91 | 6.91 | 38.4 | 265 | 1629 | 2.31 |
| 48 | 560 | 2.07 | 3.52 | 7.26 | 7.28 | 29.8 | 200 | 1611 | 2.27 |
| 49 | 600 | 1.97 | 3.53 | 7.52 | 7.57 | 28.5 | 147 | 1355 | 2.13 |
| 50 | 1120 | 2.07 | 3.59 | 7.22 | 7.43 | 24 | 149 | 1338 | 1.99 |
| 51 | n/a | n/a | n/a | n/a | n/a | n/a | n/a | n/a | 0.00 |
| 52 | 1160 | 1.95 | 3.33 | 6.51 | 6.64 | 36.8 | 219 | n/a | 0.00 |
| 53 | 1120 | 1.99 | 3.46 | 7 | 7.07 | 37.5 | 179 | n/a | 0.00 |
| 54 | 680 | 1.96 | 3.34 | 6.91 | 6.95 | n/a | n/a | n/a | 0.00 |
