# Supplementary material for: The physical profile of female cricketers: An investigation between playing standard and position
Source: PLoS One. 2024 Jun 10;19(6):e0302647. doi: 10.1371/journal.pone.0302647 (PMC11164355; doi:10.1371/journal.pone.0302647)
Supplement: Attachment — Submitted filename: Response to Reviewers_Revision 2.docx [file pone.0302647.s004.docx]

**Response to Reviewers – Version 2**

**Reviewer 2 Comments**

**Reviewer #2: Some comments have been addressed and the paper seems to me more clear; however, some others require further attention.**

The authors would like to thank the reviewer for their time and consideration in reviewing the manuscript. Please see below specific response to the comments/questions raised.

**Which version of the Declaration of Helsinki were the authors referring to?**

Thank you for your comment. Amendments have been made to the manuscript.

“The research was approved by the institutional ethics committee, in agreement with the declaration of Helsinki, version seven.”

**Did the authors intend to refer to adrenergic or androgenic substances?**

Thank you for your comment. Amendments have been made to the manuscript.

“All Participants were instructed to refrain from vigorous physical exercise, caffeine, or any adrenergic substance in the 24-hours prior to data collection, and to maintain their normal dietary habits.”

**Regarding the statistical details, I recommend including supplementary materials if the authors prefer tables or figures instead of textual descriptions. It is particularly crucial to comprehend the magnitude of these differences, especially considering that the authors opted not to provide the sample size calculation.**

Thank you for your recommendation. As noted, the statistical details (i.e., F statistic, degrees of freedom and p-value) are presented in text within the results section. The authors believe that due to the concise results section, readers can easily access and interpret these statistical details from the text. Hence, the authors do not believe that this information needs to be transferred to a table, and feel that the current presentation of statistical details is appropriate. However, if the reviewer is adamant that this change is necessary, and the editor agrees, the authors will consider this amendment to the manuscript.
